# Preparation and Properties of Polyvinylpyrrolidone/Sodium Carboxymethyl Cellulose Soluble Microneedles

**DOI:** 10.3390/ma16093417

**Published:** 2023-04-27

**Authors:** Na Qiang, Zhu Liu, Ming Lu, Yong Yang, Fangli Liao, Ying Feng, Guocong Liu, Si Qiu

**Affiliations:** School of Chemistry and Materials Engineering, Huizhou University, Huizhou 516007, China

**Keywords:** dissolving microneedle, composite material, properties

## Abstract

Transdermal drug delivery is a new means of delivering drugs through the skin to achieve therapeutic effects. Microneedles have several advantages, including low cost, easy self-administration, and high delivery efficiency. Different polymers affect the morphology, mechanical properties, and drug delivery efficiency of microneedles. To study the performance and limitations of microneedles (MNs), we prepared different ratios of polymers. MNs were fabricated from polyvinylpyrrolidone (PVP) and sodium carboxymethyl cellulose (CMC-Na) using the centrifugal molding method. Needle morphology, formability, and other properties of the polymers were evaluated to compare the performances of MNs with different ratios. PVP and CMC-Na were intermixed at different ratios with water as the solvent. The soluble MNs were prepared by mold casting. The morphology, thermodynamic properties, and crystallinity were studied using scanning electron microscopy (SEM), thermogravimetric analysis (TG), differential scanning calorimetric analysis (DSC), and X-ray diffraction (XRD). The results showed that composite microneedles have good thermal stability. Among the different compositions tested, the 10% PVP/2% CMC-Na composite microneedle demonstrated the best performance with a regular surface morphology and relatively high thermal decomposition and melting temperatures. These results indicate that microneedles with appropriate ratios of two different materials possess good formability and other properties.

## 1. Introduction

Microneedles are a new type of transdermal drug delivery technology [1,2,3] that, due to their advantages such as minimal invasiveness, safety, and efficiency, have been widely used for vaccination [4,5,6], local anesthesia [7,8], the treatment of diabetes [9,10], and the delivery of active cosmetic products [11,12]. Microneedles are composed of multiple nanoscale needle tips arranged neatly on a base. The length of microneedles is generally between 25 and 1000 μm. The thickness of the human skin’s corner layer is about 10–15 μm, so drug-loaded microneedles can penetrate the corner layer and form micron-sized channels between the epidermis and dermis. The administered drug is released through multiple micrometer pores formed on the skin’s surface. In addition, the length and shape of the microneedles are designed to allow the drug to reach the desired depth into the skin.

Microneedles can be divided into solid microneedles, hollow microneedles, coated microneedles, and soluble microneedles according to their application in the field of drug introduction [13,14]. The application of solid microneedles has been reported in the literature [15]. Solid microneedles are generally made of metal materials and non-degradable polymers [16] obtained using the casting method. They are used to deliver drugs through the skin’s corner layer because of their desirable mechanical properties [17,18]. However, residual metals and non-degradable polymers left in the skin when the needle is broken represent safety hazards to the human body. Hollow microneedles deliver drugs in the form of a microinjections. They are generally applied using a digitally controlled hollow microneedle injection system [14,19], thus achieving quantitative drug release. However, the preparation process is complex and time-consuming. Coated microneedles are prepared by the impregnation method. The drug carried by coated microneedles is directly pierced into the skin and then released because the drug is directly coated on the surface of the microneedles. However, the amount of drug that can be loaded on coated microneedles is small and is difficult to control accurately. Many researchers have turned to the field of soluble microneedles for the development of microneedle transdermal drug delivery. The advantages of soluble microneedles include excellent degradability, biocompatibility, a simple preparation method, low cost, large drug load, a wide application range, a lack of sharp waste residue, easy-to-control drug release, etc. [13,20,21]. The preparation methods for soluble microneedles include casting, hot pressing, injection molding, and mold casting.

The needle body of soluble microneedles is mainly composed of natural carbohydrates and synthetic soluble polymers. Natural carbohydrates include hyaluronic acid (HA) [22,23], chitosan (CS) [24,25], silk fibroin, etc. Synthetic soluble polymers include polyvinyl alcohol (PVA) [26], polyvinylpyrrolidone (PVP) [27], polylactic acid (PLA) [28], etc. The above materials can be divided into brittle materials and ductile materials according to their mechanical properties. Brittle materials have some mechanical strength, but brittle fracture of the tip may occur when they are used alone. The flexibility of the needle made with ductile materials is good, making the tip less easy to break. However, the mechanical properties of the tip are poor when ductile materials are used alone, and the tip cannot completely pierce the skin. Therefore, brittle materials and ductile materials are usually combined to obtain soluble microneedles with sufficient mechanical strength.

PVP is an amphiphilic polymer. It is widely used, owing to its satisfactory biocompatibility, solubility, and bio-inertia [29]. When soluble microneedles are prepared with PVP alone, the microneedle tip is brittle and easy to break. CMC-Na is a kind of material with good biocompatibility and stable water solubility [30]. Microneedles prepared with CMC-Na alone have satisfactory toughness but lack mechanical strength and deform during curing. Soluble microneedle materials with satisfactory mechanical properties can be obtained by mixing PVP and CMC-Na.

Many scientists have attempted to bring together every attribute of MNs, from their materials to their application in diverse fields. The materials used to fabricate MNs have a significant impact on drug delivery utilizing this novel technology. In this study, PVP and CMC-Na composite soluble microneedles were prepared at different ratios. The preparation of and molding process for microneedles were explored. The drying and demolding time, needle content, crystallinity, and thermal stability of microneedles were all different at different blending ratios. The properties of soluble microneedles were characterized by thermogravimetric analysis (TG), differential scanning calorimetry (DSC), scanning electron microscopy (SEM), and X-ray diffraction (XRD). The physicochemical and mechanical properties influence the drug release kinetics. This provides reference for the selection of soluble microneedle matrix materials.

## 2. Materials and Methods

### 2.1. Materials

PVP (Sinopharm Chemical Reagent Co., Ltd., Lot No. 20201029) had an *M*_n_ value of 44,000–54,000, with a K value of 27–35. CMC-Na was obtained from Zhejiang Yinuo Biotechnology Co., Ltd. (Ningbo, China). The reagents used in this experiment were purchased from Nanyuan company, Huizhou City. PVP was commercially available and of analytically pure grade. The sodium CMC-Na used in this study was of food grade.

### 2.2. Characterization

SEM: The MNs were sputter-coated with gold and visualized using a scanning electron microscope (SEM) (SEM, JSM-6380LA Analytical SEM, JEOL Ltd., Tokyo, Japan) operated at an accelerating voltage of 15 kV.

TG: The thermal decomposition temperature was investigated using a TGA 209 F1 purged with nitrogen. TG was carried out in the temperature range of 35–600 °C at a scanning rate of 10 °C/min with a gas flow rate of 20 mL/min.

DSC: The crystallization behavior of the polymers was investigated using a modulated differential scanning calorimeter (MDSC 2910, TA Instruments, New Castle, DE, USA) purged with nitrogen in the cooling and heating process. DSC was carried out in the temperature range from 0 °C to 250 °C at a scanning rate of 10 °C/min.

XRD: The crystal structure of the samples was investigated using a Rigaku Ultima IV X-ray powder diffractometer with Cu Kα radiation (λ = 1.5406 Å and 40 kV, 40 mA).

### 2.3. Methods

**Figure 1:**
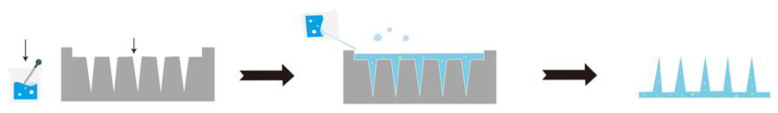
Schematic diagram of the microneedle production process.

The preparation of soluble microneedles included three steps. The preparation process is shown in Figure 1. PVP and CMC-Na were dissolved in water at different ratios. The total weight of the solute and the solvent was 10 g. The quality percentage of PVP and CMC-Na was a single variable. A series of solutions were prepared.

The solution preparation process was as follows: First, the solute was placed into a beaker. Then, water was added to the beaker by stage addition and stirred thoroughly. A large number of small bubbles were produced in the solution through stirring. Because the inclusion was formed when the outer powder came into contact with water at the initial stage of polymer dissolution, small bubbles were generated when stirred; this is consistent with what the literature reports.

It would take a long time if the solution were painted on the surface of the mold and centrifuged because the solution in the mold would be easily detached from the mold surface during centrifugation and would be difficult to control. Therefore, the bubbles had to be removed before casting the mold. There are two ways to remove bubbles in the solution. The first is to place the prepared solution into a centrifuge tube to remove the bubbles by centrifugation and then transfer the solution into the mold. The second method involves leaving the prepared solution to de-bubble prior to molding into microneedles. The viscosity of the solution was high because of the presence of CMC-Na. The centrifuge tube was not easy to clean, and part of the solution was lost during the transfer process. Therefore, the second method was used to remove the bubbles in the solution, and the microneedles were prepared by mold casting.

## 3. Results and Discussion

### 3.1. Preparation of MNs

The main preparation methods for microneedles included casting, hot pressing, injection molding, and mold casting. The mold casting method was adopted in this experiment. The prepared solution was evenly spread on the surface of the microneedle mold. The mold was placed flat in the plastic centrifuge tube, and the solution was centrifuged to fully enter the mold.

After centrifugation, the soluble microneedle mold was dried and demolded. We found that the drying time varied depending on the mass percentage of PVP in the solution. Furthermore, the drying temperature has an effect on the drying time of composite microneedles but has little effect on the molding of microneedles. The drying condition of microneedles should be observed every 10 min toward the end of the drying period. The drying temperature was set as 45 °C to facilitate timely observation of microneedle formation and timely removal of microneedles for the demolding operation.

PVP and CMC-Na were combined in different mass percentages to obtain the best microneedles with satisfactory formability and high hardness that were not easy to fracture. The percentage of prepared soluble microneedles is shown in Table 1.

The needle content and molding type of microneedles varied depending on the mass percentage of the microneedle materials. Therefore, the experimentally obtained soluble microneedles were filtered prior to the characterization study. The effect of the different mass percentages of the matrix materials on the microneedles’ performance was investigated. The composite microneedles containing 10%PVP/2%CMC-Na, 20%PVP/3%CMC-Na, and 30%PVP/3%CMC-Na were characterized to investigate the effects of matrix materials containing different mass percentages on microneedle performance.

### 3.2. Morphology of the MNs

**Figure 2:**
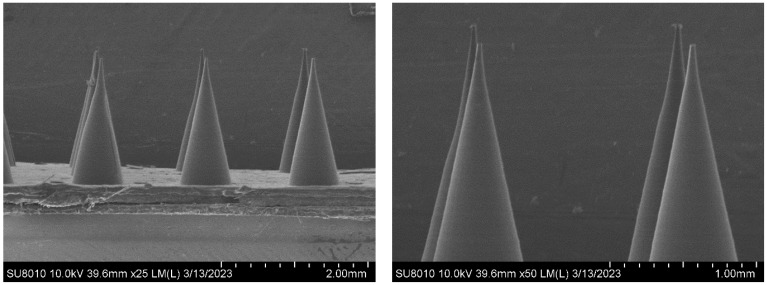
SEM image of composite microneedles (10%PVP/2%CMC-Na).

**Figure 3:**
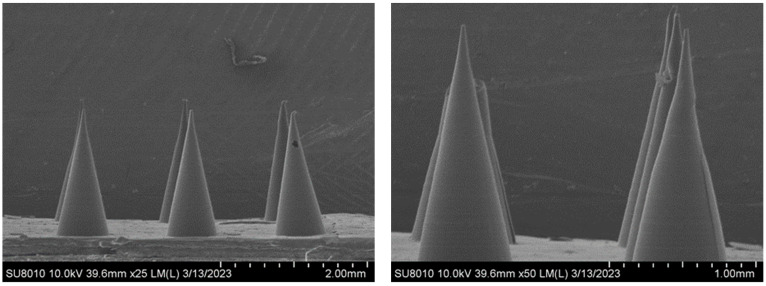
SEM image of composite microneedles (20%PVP/3%CMC-Na).

**Figure 4:**
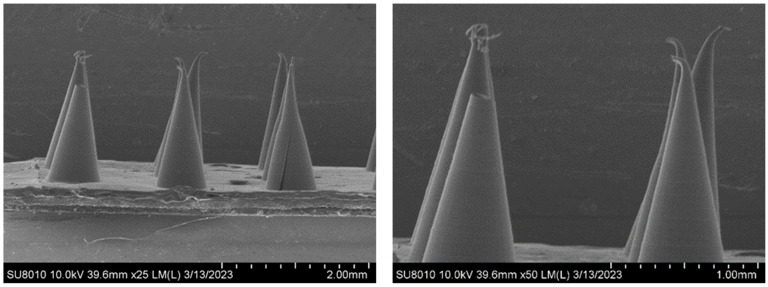
SEM image of composite microneedles (30%PVP/3%CMC-Na).

The micromorphology of the composite microneedles with different mass percentages was observed under a scanning electron microscope (SEM). Figure 2 shows a SEM image of the 10%PVP/2% CMC-Na composite microneedle. The composite microneedles were conical and uniform in length, with a smooth surface and satisfactory shape. Figure 3 shows a SEM image of the 20%PVP/3% CMC-Na composite microneedle. The upper part of the microneedle body was inclined, with a tip-curling phenomenon. However, the surface of the microneedle body was smooth and regular. There were two main reasons for this phenomenon. First, the drying time was insufficient during the process of drying and demolding. Second, PVP and CMC-Na are hydrophilic. The microneedles failed to maintain a dry environment during the reserve process, and the needle body was squeezed after absorbing water [31]. Figure 4 shows a SEM image of the 30%PVP/3% CMC-Na composite microneedle. The surface of the composite microneedles at this mass percentage was rough. However, the overall regularity was satisfactory, and the needle body was not tilted significantly. In summary, the surface smoothness and overall regularity of the composite microneedles varied depending on the mass percentage. However, the needle bodies of the microneedles were conical, and their structures were relatively regular.

### 3.3. Mechanical Properties of MNs

**Figure 5:**
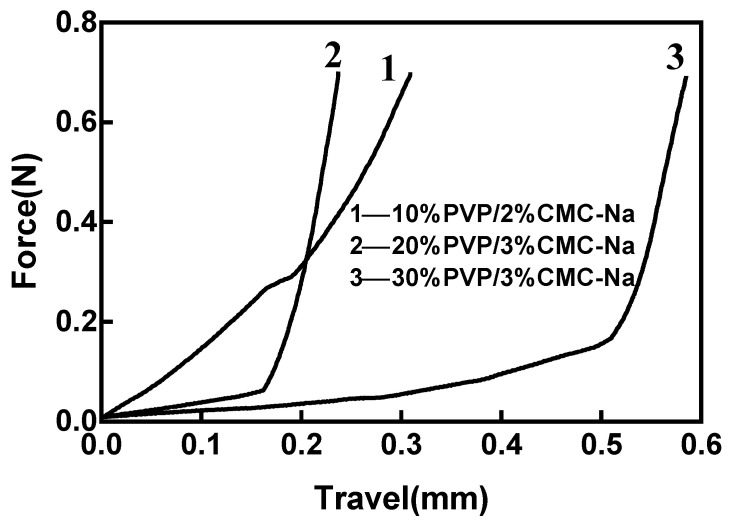
Force–travel curve of MNs with different ratios (1–10%PVP/2%CMC-Na; 2–20%PVP/3%CMC-Na; 3–30%PVP/3%CMC-Na).

Figure 5 shows the force–travel curves of the three microneedles, demonstrating significant differences in the mechanical properties of the three microneedles on the basically identical curve surface. The higher the slope of the force–displacement curve, the higher the mechanical strength, which is slightly higher for the 10%PVP/2% CMC-Na than the other two microneedles. The yield strength represented by the inflection point of the curve is more representative of the puncture performance of the microneedles. Studies have shown that a microneedle only needs to withstand 0.2–0.3 N of force to penetrate the skin; clearly, the 10%PVP/2% CMC-Na sample can meet this standard of mechanical strength [32].

### 3.4. Thermodynamic Properties of MNs

**Figure 6:**
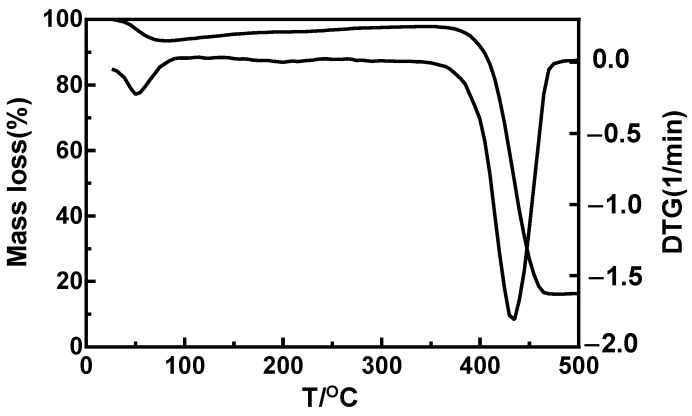
TG and DTG curve of pure PVP.

As shown in Figure 6, there were two decomposition processes for PVP in the TG and differential thermal gravity (DTG) curves. The TG curve shows that PVP began to lose weight at 38.2 °C, with weight loss amounting to 2.3%. The process was not obvious in the TG curve, representing water decomposition. The thermal decomposition temperature of PVP was 399.8 °C, which is close to the data reported in the literature [33]. PVP has only one decomposition process and has good thermal stability, with 18.5% retained at 500 °C.

**Figure 7:**
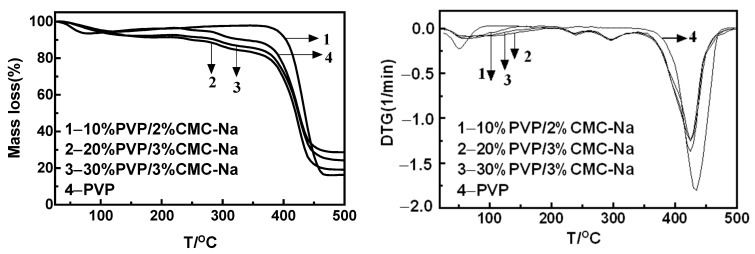
TG and DTG curve of composite microneedles and PVP with different ratios (1−10%PVP/2%CMC-Na; 2−20%PVP/3%CMC-Na; 3−30%PVP/3%CMC-Na; 4−PVP).

As shown in Figure 7, the decomposition temperature varied among the composite microneedles, with three weight loss stages of composite microneedles according to the TG and DTG curves. The first two weight loss stages were not obvious; this was verified as the weight loss stage of CMC-Na in the literature [34]. Therefore, relevant analysis was mainly carried out at the last weight loss stage. Combined with the TG and DTG curves of PVP shown in Figure 6, it can be seen that the last weight loss stage was the thermal decomposition process of PVP. According to the curves presented in Figure 7, the decomposition temperature of the composite microneedles containing 10%PVP/2% CMC-Na was 379.8 °C. The decomposition temperature of the 20%PVP/3% CMC-Na composite microneedle was 369.8 °C. The decomposition temperature of the 30%PVP/3% CMC-Na composite microneedle was 374.8 °C. The thermal decomposition temperature of the composite microneedles varied depending on the mass percentage. Furthermore, the composite microneedles had a lower thermal decomposition temperature than that of the PVP polymer. The 10%PVP/2% CMC-Na composite microneedle had the highest thermal decomposition temperature, resulting in the best thermal stability performance.

**Figure 8:**
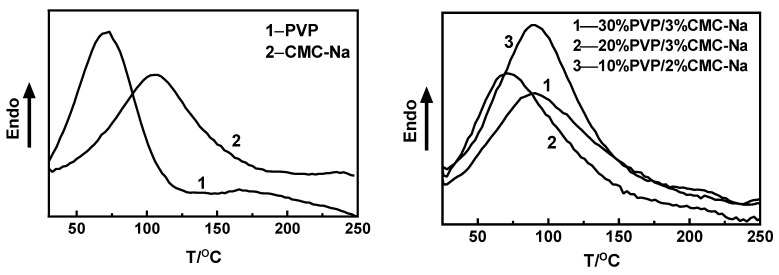
DSC curve of PVP, CMC-Na and composite microneedles with different ratios.

As shown in Figure 8, PVP and CMC-Na have an upward melting peak at 70 °C and 105 °C, respectively. Figure 8 shows that the 30%PVP/3% CMC-Na, 20%PVP/3% CMC-Na, and 10%PVP/2% CMC-Na composite microneedles have melting peaks at 89 °C, 72 °C, and 91 °C, respectively. The melting temperature of the composite microneedles was higher than that of PVP but lower than that of CMC-Na. The melting temperature of the 10%PVP/2% CMC-Na composite microneedle was the highest. The melting peak distribution of the soluble microneedles was wide, so the crystallization of the composite microneedles was not uniform. The integrity of the crystal was destroyed. The mass percentage of the mixture of PVP and CMC-Na has an effect on the crystallization mode of the composite microneedles.

### 3.5. X-ray Diffractometry of MNs

**Figure 9:**
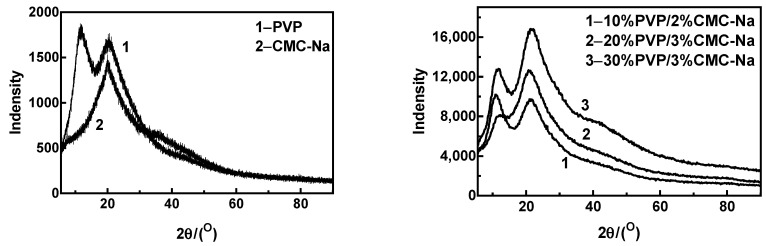
XRD curve of PVP, CMC-Na, and composite microneedles with different ratios.

According to the XRD images of the composite microneedles presented in Figure 9, the composite microneedles with different mass percentages had two prominent peaks. However, the two peaks were not sharp, which indicates that the crystalline phase in the sample was very incomplete. The XRD curve of the 10%PVP/2% CMC-Na composite microneedle showed strong diffraction peaks at 10.84° and 21.10°. According to the XRD curve of the 20%PVP/3% CMC-Na composite microneedle, the main diffraction peak occurred at 20.86°, and the peak intensity was weak at 12.58°. The XRD curve of the 30%PVP/3% CMC-Na composite microneedle shows strong diffraction peaks at 11.54° and 21.68°. As can be seen from the figure, the positions of the main diffraction peaks of composite microneedles with different mass percentages were basically unchanged, but the intensity of the diffraction peaks changed significantly. Therefore, the crystal patterns of the composite microneedles with different mass percentages were not significantly different, but the crystal structure changed to some extent.

## 4. Conclusions

Polymeric microneedles (MNs) are a technology for delivering small chemical molecules into large complex biotherapeutics. The successful development of polymer MNs relies on the type of polymer used (one polymer or mixture of polymers), the design, and mechanical strength of the MNs. Fundamental studies are required. In the current study, PVP and CMC-Na were selected for the preparation of composite soluble microneedles due to their demonstrated biocompatibility and complementary mechanical properties. The optimization of the formula and preparation process resulted in the production of the soluble microneedles via a step-by-step mold casting method. The analysis of the surface morphology via scanning electron microscopy (SEM) revealed that the prepared microneedles had a smooth needle body and a well-defined tip morphology, with no observable pores or gaps on the surface. The TG and DTG analysis indicated that the mass percentage of the composite microneedles, centrifugation time, drying time, and demolding time had an impact on the needle content and molding ability of the microneedles. The thermal decomposition temperature and melting temperature of the microneedles were found to vary with the mass percentage of the composite. The results showed that the composite microneedles have good thermal stability. Among the different compositions tested, the 10% PVP/2% CMC-Na composite microneedle demonstrated the best performance with a regular surface morphology and relatively high thermal decomposition and melting temperatures. In summary, composite soluble microneedles were successfully prepared and characterized. Therefore, there is great potential for the development of composite materials that ensure appropriate drug loads and sustained release. These results may promote the prospective application of composite microneedles as a suitable sustained-release drug delivery system. Future developments in MNs will need to consider the characteristics of the target disease and the target tissue. This will determine the MNs’ design, the type of drug that can be used, and the amounts for effective delivery into the target tissue.

## Figures and Tables

**Table 1:** Soluble microneedle preparation percentage and results.

Microneedle Material (Mass Percentage)	Centrifugal Condition	Drying Temperature	Drying Time	Molding Microneedle Content	Microneedle Formability
10%PVP 2%CMC-Na	2000 r/min (3 min) +2500 r/min (2 min)	45 °C	120 min	85%	√
15%PVP 3%CMC-Na	2000 r/min (3 min) +2500 r/min (2 min)	45 °C	150 min	70%	×
15%PVP 2%CMC-Na	2000 r/min (3 min) +2500 r/min (3 min)	45 °C	100 min	20%	×
20%PVP 3%CMC-Na	2500 r/min (5 min) +2000 r/min (3 min)	45 °C	180 min	100%	√
25%PVP 3%CMC-Na	2500 r/min (5 min) +2000 r/min (5 min)	45 °C	200 min	100%	×
30%PVP 3%CMC-Na	2500 r/min (5 min) +2000 r/min (5 min)	45 °C	240 min	100%	√
